# A computational method for genotype calling in family-based sequencing data

**DOI:** 10.1186/s12859-016-0880-5

**Published:** 2016-01-16

**Authors:** Lun-Ching Chang, Bingshan Li, Zhou Fang, Scott Vrieze, Matt McGue, William G. Iacono, George C. Tseng, Wei Chen

**Affiliations:** Division of Cancer Treatment and Diagnosis, National Cancer Institute, Bethesda, MD 20892 USA; Department of Molecular Physiology & Biophysics, Vanderbilt University Medical Center, Nashville, TN 37232 USA; Department of Biostatistics, University of Pittsburgh, Pittsburgh, PA 15261 USA; Department of Psychology & Neuroscience, Institute for Behavioral Genetics, University of Colorado, Boulder, CO 80309 USA; Department of Psychology, University of Minnesota, Minneapolis, MN 55455 USA; Division of Pulmonary Medicine, Allergy and Immunology, Children’s Hospital of Pittsburgh of UPMC, Pittsburgh, PA 15224 USA

**Keywords:** Family-based sequencing, Genotype calling, Hidden Markov model

## Abstract

**Background:**

As sequencing technologies can help researchers detect common and rare variants across the human genome in many individuals, it is known that jointly calling genotypes across multiple individuals based on linkage disequilibrium (LD) can facilitate the analysis of low to modest coverage sequence data. However, genotype-calling methods for family-based sequence data, particularly for complex families beyond parent-offspring trios, are still lacking.

**Results:**

In this study, first, we proposed an algorithm that considers both linkage disequilibrium (LD) patterns and familial transmission in nuclear and multi-generational families while retaining the computational efficiency. Second, we extended our method to incorporate external reference panels to analyze family-based sequence data with a small sample size. In simulation studies, we show that modeling multiple offspring can dramatically increase genotype calling accuracy and reduce phasing and Mendelian errors, especially at low to modest coverage. In addition, we show that using external panels can greatly facilitate genotype calling of sequencing data with a small number of individuals. We applied our method to a whole genome sequencing study of 1339 individuals at ~10X coverage from the Minnesota Center for Twin and Family Research.

**Conclusions:**

The aggregated results show that our methods significantly outperform existing ones that ignore family constraints or LD information. We anticipate that our method will be useful for many ongoing family-based sequencing projects. We have implemented our methods efficiently in a C++ program FamLDCaller, which is available from http://www.pitt.edu/~wec47/famldcaller.html.

**Electronic supplementary material:**

The online version of this article (doi:10.1186/s12859-016-0880-5) contains supplementary material, which is available to authorized users.

## Background

Next generation sequencing technologies have greatly aided in comprehensively identifying common variants (e.g. minor allele frequency (MAF) >1 %) and rare variants (MAF <1 %), helping researchers understand genetic coding and discover thousands of disease-susceptible variants. For example, the recent 1000 Genomes Project has provided characterization of human genome sequence variation, aiding in understanding the relationship between genotype and phenotype [1, 2]. Sequencing studies in families have unique advantages and strengths in controlling population stratification, studying parent-of-origin effects, identifying rare causal variants and detecting *de novo* mutations [3–8]. Sequencing has also proven successful in studying Mendelian disorders in families [6, 9]. Numerous family-based sequencing projects (often in the design of large number of trios/nuclear families or a mixture of unrelated individuals and small families) have been carried out or launched to study complex diseases [10–14]. Many ongoing sequencing projects include nuclear families (two parents with one or more offspring) or multi-generational families. Existing approaches include methods that either focus on single sites, split pedigree into trios, or treat all sequenced samples as unrelated individuals. Among the few existing methods for genotype calling of family-based sequence data, most methods consider family constraints at each marker [15, 16] independently. The other methods consider LD information but ignore family constraints. Recently, Chen et al. proposed a method of genotype calling method by considering family structure in parent-offspring trios and showed the method can achieve more accurate genotype calls in great amounts as compared with the one without considering the family structure (reduce genotype calling error rate by 50 %) [17]. However, to our knowledge, there is no existing method that jointly models family constraints and LD pattern in complex pedigree (nuclear and extended families).

In this paper, motivated by the previous methods, we describe a novel method for genotype calling and phasing in nuclear and extend families. The purpose of the present paper is twofold. The first is to extend our previous method from analyzing trios to nuclear families or families with multi-generations in a computationally efficient manner. Here we focus on developing the procedure by looping all possible parent-offspring trios to update the probability of observed genotype given the true genotype simultaneously, which is a pivotal step in a hidden Markov model (HMM). Through two simulated studies, which are with/without alignment and experimental errors, we evaluate the performance by using the genotype error calling rate and phasing error (as haplotypes are provided), and we show that incorporating more offspring within family (or complex family with multiple generations) can have more accurate genotype calls than trios only, especially in low to modest depth in sequencing data. Secondly, the method can be extended to incorporate external reference panels for analyzing sequencing dataset including a small number of samples. This is motivated by many pilot projects, which often include a limited number of samples (e.g. one or two trio) and LD information is not available in the study population. External reference panels (e.g. the 1000 Genomes Project) will be useful in this scenario to facilitate genotype calling and phasing if the LD pattern in the study population is well captured. Through both simulated and real studies, we show that our methods outperform the existing methods that do not use LD information or ignore the complex family constraints.

## Methods

SNP discovery and genotype calling are two key steps for downstream analyses after massive reads are generated by high-throughput sequencing machines [18–20]. In this paper, we only focus on refining genotypes after polymorphic sites are discovered. We only focus on bi-allelic markers and start with observed data *P*(*R*_*i*_|*G*_*i*_), the likelihood of observed read *R*_*i*_ given an underlying true genotype *G*_*i*_ for each position *i* from all candidate variants. For each site, the number of reference and alternative alleles are counted from the reads that cover the study site, then $P\left({R}_i\Big|{G}_i\right)$ is routinely calculated by conventional tools (e.g. SAMtools) using various models such as a binomial distribution [21]. For example, the likelihoods *P*(*R*_*i*_|*G*_*i*_) by assuming independent error can be written as $P\left({R}_i=\left(\mathbf{B},\ \mathbf{E}\right)\Big|{G}_i=\left\{1,\ 1\right\}\right)={\varPi}_j{\left(1-{e}_j\right)}^{I\left({b}_j=1\right)}{\left(\frac{1}{3}{e}_j\right)}^{I\left({b}_j\ne 1\right)}$ for homozygous call “1/1” and $P\left({R}_i=\left(\mathbf{B},\ \mathbf{E}\right)\Big|{G}_i=\left\{1,\ 2\right\}\right)={\varPi}_j\left\{\frac{1}{2}{\left(1-{e}_j\right)}^{I\left({b}_j=1\right)}{\left(\frac{1}{3}{e}_j\right)}^{I\left({b}_j\ne 1\right)}+\frac{1}{2}{\left(1-{e}_j\right)}^{I\left({b}_j=2\right)}{\left(\frac{1}{3}{e}_j\right)}^{I\left({b}_j\ne 2\right)}\right\}$ for heterozygous call “1/2”, where **B** and **E** denote the vectors of base calls and corresponding error probabilities. *P*(*R*_*i*_|*G*_*i*_) serves as input data in our model.

### HMM approach to describe chromosome mosaics

Li and Stephens indicated that the haplotypes of each individual can be described as imperfect mosaics of other haplotypes in the sample by using hidden Markov model (HMM) [22], and this approach has been successfully applied to genotype imputation and haplotype reconstruction [23–25]. This approach has also been used in genotype calling for sequence data. In this section, we briefly reviewed the HMM method to model unrelated samples for the sequence data. First, we sampled an allele from each individual haplotype in reference panels consistent with observed data at each position. Second, we used HMM method to update the haplotype for each individual, describing the pair of haplotypes as an imperfect mosaic of other reference panels.

Here we will describe the HMM model to show how we update the haplotypes of each individual conditional on all the other samples’ haplotype estimates. We denote the probability of an underlying truth genotype ${G}_i$ given the mosaic state *S*_*i*_, *P*(*G*_*i*_|*S*_*i*_). The function *T*(*S*_*i*_) was defined as the number of different alleles for genotype *G*_*i*_ = {0, 1, 2}. So *P*(*G*_*i*_|*S*_*i*_) was defined by

1 $$ \left\{\begin{array}{cc}\hfill {\left(1-{\varepsilon}_i\right)}^2\hfill & \hfill \left[T\left({S}_i\right)=0\ \mathrm{or}\ T\left({S}_i\right)=2\right]\ \mathrm{and}\ T\left({S}_i\right)=T\left({G}_i\right)\hfill \\ {}\hfill {\varepsilon}_i\left(1-{\varepsilon}_i\right)\hfill & \hfill \left[T\left({S}_i\right)=0\ \mathrm{or}\ T\left({S}_i\right)=2\right]\ \mathrm{and}\ \left|T\left({S}_i\right)-T\left({G}_i\right)\right| = 1\hfill \\ {}\hfill {\varepsilon_i}^2\hfill & \hfill \left[T\left({S}_i\right)=0\ \mathrm{or}\ T\left({S}_i\right)=2\right]\ \mathrm{and}\ \left|T\left({S}_i\right)-T\left({G}_i\right)\right| = 2\hfill \\ {}\hfill {\left(1-{\varepsilon}_i\right)}^2+{\varepsilon_i}^2\hfill & \hfill T\left({S}_i\right)=1\ \mathrm{and}\ T\left({S}_i\right)=T\left({G}_i\right)\hfill \\ {}\hfill 2{\varepsilon}_i\left(1-{\varepsilon}_i\right)\hfill & \hfill T\left({S}_i\right)=1\ \mathrm{and}\ T\left({S}_i\right)\ne T\left({G}_i\right)\hfill \end{array}\right. $$

where *ε*_*i*_ is the cumulative effects of mutation and gene conversion (we called it mosaic error rate here) at marker *i*. Then we can calculated the emission probability of *P*(*R*_*i*_|*S*_*i*_) as:

2 $$ P\left({R}_i\Big|{S}_i\right)={\displaystyle {\sum}_{G_i}P\left({R}_i\Big|{G}_i\right)\times P\left({G}_i\Big|{S}_i\right)} $$

Then the transition probability *P*(*S*_*i* + 1_|*S*_*i*_) in the HMM was defined by

$$ P\left({S}_{i+1}=\left(w,v\right)\Big|{S}_i=\left(x,y\right)\right) $$

3 $$ =\left\{\begin{array}{cc}\hfill \frac{\theta_i^2}{H^2}\hfill & \hfill w\ne x\ \mathrm{and}\ y\ne v\hfill \\ {}\hfill \frac{\left(1-{\theta}_i\right){\theta}_i}{H}+\frac{\theta_i^2}{H^2}\hfill & \hfill \left\{w\ne x\ \mathrm{and}\ y=v\right\}\mathrm{or}\ \left\{w=x\ \mathrm{and}\ y\ne v\right\}\hfill \\ {}\hfill {\left(1-{\theta}_i\right)}^2+\frac{2\left(1-{\theta}_i\right){\theta}_i}{H}+\frac{\theta_i^2}{H^2}\ \hfill & \hfill w=x\ \mathrm{and}\ y=v\hfill \end{array}\right. $$

where *θ*_*i*_ is the mosaic transition rate from position *i* to position *i + 1*, and *H* is the number of haplotypes in the reference panel. Our goal is to calculate $P\left({G}_i\Big|\mathbf{R}\right)$, the probability of a genotype at position *i* conditional on all sequence reads:

4 $$ P\left({G}_i\Big|\mathbf{R}\right)={\displaystyle {\sum}_{S_i}P\left({G}_i\Big|{S}_i\right)\times P\left({S}_i\Big|\mathbf{R}\right)} $$

by looping all possible state ${S}_i$. Baum’s forward-backward algorithm was used to calculate $P\left({S}_i\Big|\mathbf{R}\right)$ and $P\left({G}_i\Big|\mathbf{R}\right)$ [26].

The model we described was based on the unrelated individuals. Chen et al. (2013) proposed a computationally efficient genotyping method of joint modelling for trios by considering both LD and the constraints of Mendelian inheritance [17]. Suppose ${R}_f$, ${R}_m$ and ${R}_c$ are observed genotype calls; ${G}_f$, ${G}_m$ and ${G}_c$ are underlying truth genotype calls and the corresponding genotype likelihoods are $P\left({R}_f\Big|{G}_f\right)$, $P\left({R}_m\Big|{G}_m\right)$ and $P\left({R}_c\Big|{G}_c\right)$ for the father, mother and child within a trio, respectively. For updating the haplotypes from a trio in each iteration at position *i*, they first update the paternal haplotypes by sampling a mosaic state ${S}_{f(i)}$, then the emission probability can be replaced to

5 $$ P\left({\overline{R}}_i\Big|{S}_{f(i)}\right)={\displaystyle {\sum}_gP\left({\overline{R}}_i\Big|{G}_f=\mathrm{g}\right)\times P\left({G}_f=\mathrm{g}\Big|{S}_{f(i)}\right)} $$

where ${\overline{R}}_i$ are observed genotype calls of ${R}_{f(i)}$, ${R}_{m(i)}$ and ${R}_{c(i)}$ within a trio at position *i*. So the genotypes likelihood within a trio at position *i* conditional on father’s genotype ${G}_f=g$ is $P\left({\overline{R}}_i|{G}_f=g\right)=P\left({\overline{R}}_i,\;{G}_f=g\right)/P\left({G}_f=\mathrm{g}\right)$ =${\sum}_{g_m}P\left({R}_f\Big|{G}_f=\mathrm{g}\right)\times P\left({R}_m\left|{G}_m={g}_m\right)\times P\left({R}_c\Big|{G}_c= transmitt\left({g}_f,{g}_m\right)\right)\times P\Big({G}_m={g}_m\right)$, where the $transmitt\left({G}_f,{G}_m\right)$ is the function which returns the genotype of child conditional on gnoetypes ${G}_f$ and ${G}_m$. Second, they updated the maternal haplotype conditional on the sampled paternal genotype updated from the first step, thus $P\left({\overline{R}}_i|{S}_i,{G}_f={g}_f\right)={\sum}_gP\left({\overline{R}}_i\Big|{G}_m=g,{G}_f={g}_f\right)\times P\left({G}_m=g\Big|{S}_i\right)$, and the final expression of a trio can be written as $P\left({\overline{R}}_i\Big|{G}_m={g}_m,{G}_f={g}_f\right)=P\left({R}_f\Big|{G}_f={g}_f\right)\times P\left({R}_m\Big|{G}_m={g}_m\right)\times P\left({R}_c\right|{G}_c= transmitt\left({g}_f,{g}_m\right)$. By updating each parent at a time in each iteration can greatly reduce the computational cost without losing the genotype accuracy (5 to 10 %).

### Procedure for modeling complex family

Chen et al. [17] proposed a strategy for parent-offspring trios with computational efficient modeling of LD and the constraint due to Mendelian inheritance. They showed that the method can greatly increase the accuracy of genotype calling. We extended their proposed algorithm to handle complex family by looping all possible trios across each family. Consistent with their paper, we denote ${R}_{fk}$, ${R}_{mk}$ and ${R}_{ck}$ as the read data from *k*-th possible trio loop within a nuclear family. ${G}_{fk}$, ${G}_{mk}$ and ${G}_{ck}$ as the underlying truth genotype for the father, mother and child, and the genotype likelihoods are denoted by $P\left({R}_{fk}\Big|{G}_{fk}\right)$, $P\left({R}_{mk}\Big|{G}_{mk}\right)$ and $P\left({R}_{ck}\Big|{G}_{ck}\right)$. The procedure for each iteration is described below:

I. At position *i*, we randomly select a child in family and corresponding parents, denoted by ${\overline{R}}_{i1}=\left({R}_{f(i)1},{R}_{m(i)1},{R}_{c(i)1}\right)$
II. First update parental haplotypes by sampling a mosaic state S_*f*(*i*)*1*_ for father, then the emission probability can be written as $P\left({\overline{R}}_{i1}\Big|{S}_{f(i)1}\right)={\displaystyle {\sum}_gP\left({\overline{R}}_{i1}\Big|{G}_{f(i)1}=g\right)\times P\left({G}_{f(i)1}=g\Big|{S}_{f(i)1}\right)},$ and $P\left({\overline{R}}_{i1}\Big|{G}_{f(i)1}=g\right)=\frac{P\left({\overline{R}}_{i1},{G}_{f(i)1}=g\right)}{P\left({G}_{f(i)1}=g\right)}={\displaystyle {\sum}_{g_m}P\left({R}_{f(i)1}\Big|{G}_{f(i)1}=g\right)\times P\left({R}_{m(i)1}\Big|{G}_{m(i)1}={g}_m\right)\times P\left({R}_{c(i)1}\Big|{G}_{c(i)1}=\mathrm{transmit}\left({g}_f,{g}_m\right)\right)},$ where transmit (*G*_*f*_, *G*_*m*_) returns the genotype for child conditional on ordered parental genotypes *G*_*f*_ and *G*_*m*_
III. Update maternal haplotypes at position *i* conditional on the sampled genotype for the first parent. Thus:
  $$ P\left({\overline{R}}_{i1}\Big|{S}_{i1},{G}_{f(i)1}={g}_f\right)={\displaystyle {\sum}_gP\left({R}_{f(i)1}\Big|{G}_{f(i)1}={g}_f\right) \times P\left({R}_{m(i)1}\Big|{G}_{m(i)1}{g}_m\right)\times P\left({R}_{c(i)1}\Big|{G}_{c(i)1}=\mathrm{transmit}\left({g}_f,{g}_m\right)\right)} $$
IV. Randomly select second child (${R}_{c(i)2}$) and corresponding parents updated from previous trio loop, and repeat step I – step III until all children (${R}_{c(i)k},\;k=1,2,\dots, {n}_l\;\mathrm{where}\;{n}_l\;\mathrm{is}\;\mathrm{number}\;\mathrm{of}\;\mathrm{children}\;\mathrm{in}\;\mathrm{family}\;l$) are all used within each family.
V. Update next family and repeat step I – step IV until all families are iterated.

**Fig. 1:**
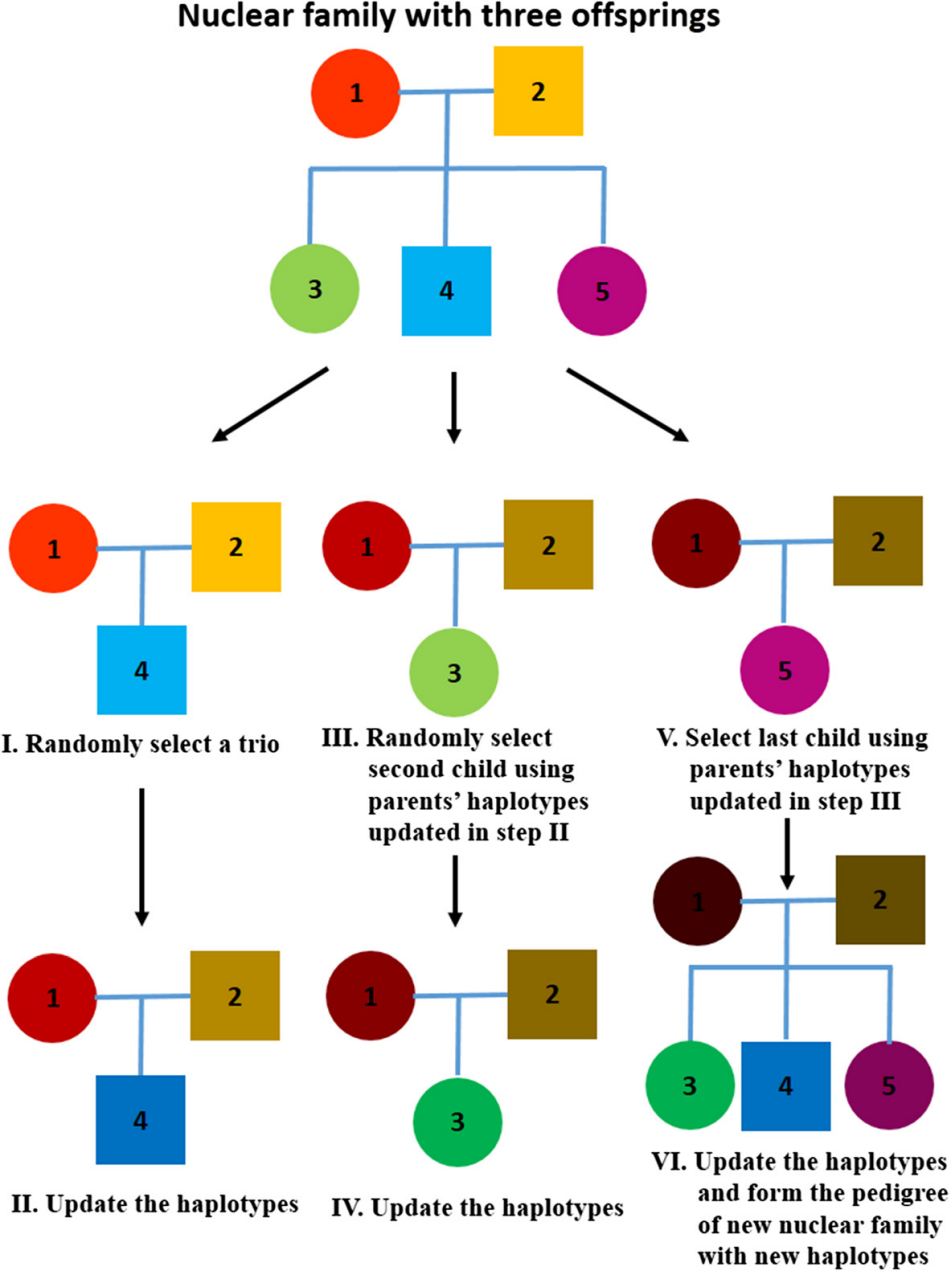
The example of updating haplotypes in a nuclear family with three offspring for each iteration. In step I, the child #4 was randomly selected to form a trio, and then we updated all three haplotypes in step II. The child #3 was randomly selected in step III and form a new trio with updated haplotypes of parents updated in step II. We repeated the same procedure until all children were selected (step IV to VI)

In each iteration, we considered all combination of the order of selecting children in each family for 100 times in the simulation study. Each round of update generates new ordered haplotypes for each family (can be unrelated individual, parent-offspring trio, nuclear family or family with multiple generations), and the consensus haplotype was generated by assigning the most frequently sampled allele at each position. Figure 1 illustrates the example of updating haplotypes for each iteration in a nuclear family with three offspring. For each iteration, we randomly selected one offspring to form a trio, and updated the haplotypes of parents and offspring (step II. and III. of the procedure). We then randomly selected second offspring to form a trio with parents’ haplotypes updated from previous step, and repeated step II and III until all possible trios were looped in each family. This method can also be applied to multi-generational family in a similar manner by looping through all offspring in a random order at each iteration.

### Use of phased reference panels

Public reference panels (e.g. 1000 Genomes Project and HapMap Project) can provide extra LD information for genotype calling and have been successful in facilitating imputation. For genotyping sequence data, most existing software do not use the information from reference panels or have to recall genotypes of reference panels together with study samples. Our method and implementation can incorporate phased reference panels efficiently into our genotype calling procedure. It has two advantages: (1) we will be able to call a small number of sequenced families/individuals using LD information from a similar population with phased haplotypes available and (2) the computation will be efficient because we don’t have to call all individuals but only sequenced individuals. This approach is particularly useful for sequencing studies with a small sample size and a reference panels from the similar population.

### Simulated data

In the first simulation scheme we considered 80 nuclear families, with each family having two founders and four offspring. To be realistic, we generated 12 regions with 1 Mb length of haplotypes (2,845,360 sites in average), with each region containing 10,000 haplotypes generated from a coalescent model to mimic the LD pattern, population demographic history, and local recombination rates of European ancestry samples [27]. We randomly sampled haplotypes for founders in each family and simulated the Mendelian transmission for the haplotypes of offspring. The short reads were simulated by assuming depth at each site followed a Poisson distribution and were defined per-based sequencing error rate. Each sample was sequenced at depth 2x, 6x and 10x by assuming per base error rate of 0.01 (Phred scaled base quality of Q20). In order to compare with the “TrioCaller” software proposed in Chen et al. [17], we considered the following procedure when calling genotypes in each nuclear family: we selected first child to form into a trio and treated other three children as unrelated subjects, which are the results of “TrioCaller”, and we then included the second child into consideration at a time until all children were used.

In the second simulation scheme, we further considered sequencing and alignment errors using the 1000 Genomes Project (1000GP) data. We simulated founders’ entire genomes by randomly selecting a pair of haplotypes from the 1000GP CEU population (March 2012 Phase 1 release). For non-founders, we simulated cross-overs in the parental haplotypes based on the genetic map in the HapMap data, and then generated offspring genotypes by randomly selecting one haplotype from each parents. We then simulated paired-end 100 bp reads according to Poisson distribution on the genome, with a mean insertion size of 400 bp and a standard deviation of 50 bp, and a sequencing error rate of 0.01 per base. We used BWA to align simulated reads to the reference of hg19 and carried out standard procedure for variant calling using Genome Analysis Toolkit (GATK) [28], including indel-realignment and base quality realignment. The list of known indels from 1000GP was provided to GATK for re-alignment prior to variant calling in different depths 5x, 10x, 20x and 30x with 3,005,070 sites on chromosome 1. There are five families, and each family has 14 members (see pedigree in Additional file 1: Figure S1). We considered the simulation settings similar to our first simulation scheme: we selected nuclear family (parents and three offspring) in each big family, then we selected first child to form into a trio and treated another two children as unrelated subjects, and included the second child into consideration at a time until all children were used. In addition, we also selected complex family with three generations from each big family.

Next, we investigate if the reference panels can help increase genotyping accuracy. We designed a simulation study by considering 2, 3 and 4 parent-offspring trios with depth 2x, 6x and 10x with per-base error rate of 0.01(Q20). For reference panels, we considered 10, 20, 40 and 60 founders from the 1000 Genome Project.

### Evaluation criteria

First, we evaluated the performance of genotype calls using *genotype mismatch rate* between genotypes estimated by our proposed algorithm and surrogated gold standard genotypes from simulated data, especially in heterozygous sites, which are more sensitive cases in genotype accuracy. Second, we calculated *switch error* that is defined as the number of *switched alleles* between estimated haplotypes by our proposed algorithm, and haplotypes from simulated data to evaluate the haplotyping accuracy. Last, we evaluated *Mendelian error* by calculating the number of incompatible alleles between each offspring and corresponding parents.

## Results

### Overall performance of genotype accuracy

**Fig. 2:**
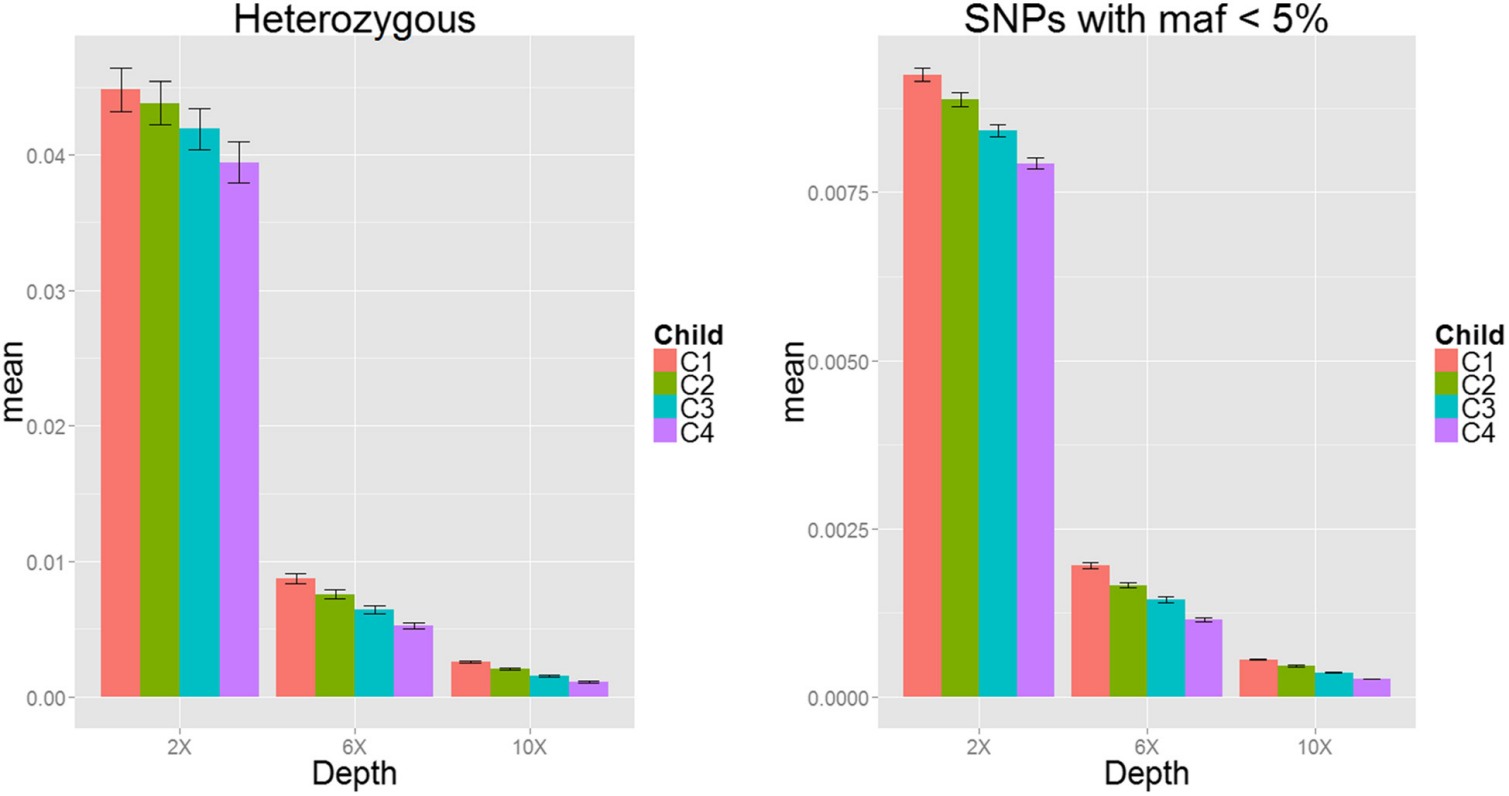
Genotype mismatch rate and standard errors of heterozygous calls and SNPs with MAF <5 % (Simulation I). The proportion of genotype mismatch rate for heterozygous SNPs (left) and SNPs with minor allele frequency (MAF) <5 % (right) with sequencing coverage of 2x, 6x and 10x and bases with Phred-scaled quality Q20 (1 % error per-based rate). (C1: trios; C2: nuclear families of two offspring; C3: nuclear families with three offspring and C4: nuclear families of four offspring.)

We evaluated the performance of our proposed algorithm for genotype calling method in simulation studies and real data analysis. We have two goals: (1) extend the existing method for analyzing trio-based data sets to handle complex family with multiple offspring and/or generations and (2) propose a function to analyze a small number of family-based samples incorporating the external reference panels, such as subjects from 1000 Genomes Project. For goal one, we considered two simulation studies with/without considering alignment and experimental errors. We first evaluated the genotype accuracy when adding more offspring in each family. Figure 2 shows the mean of the genotype mismatch rate of heterozygous calls and SNP with minor allele frequency (MAF) <5 % summarized from twelve simulated haplotypes. It shows the clear pattern that adding more offspring per family can reduce the genotype mismatch rate (see also Additional file 1: Table S1), especially in low depth (2x). The genotype mismatch rate of heterozygous calls can be reduced from 4.5 to 4.38 % to 4.18 to 3.94 % when one, two, three and all four offspring were considered, respectively. Sequencing depth also contributed to genotype accuracy: as 80 trios and 240 unrelated samples were sequenced, the genotype mismatch rates of heterozygous calls reduced from 4.48 to 0.875 % to 0.257 % as depth increase from 2x to 6x to 10x. The advantage of our proposed method makes it clear that adding more offspring can achieve more accurate genotype calls, especially at low sequencing depths. In addition, we have performed additional analysis to compare our result with the latest version of Beagle, which takes pedigree for genotype calling. We found that our proposed algorithm for genotype calling performs better than the results from Beagle (see Additional file 1: Table S1) in simulated scenarios. We performed Wilcoxon rank sum test to test the mean genotype mismatch rate of multiple offspring in the unclear family compared with the result from “TrioCaller”. For heterozygous calls at depth 2x, the p values are 0.221, 0.099 and 0.016 as including 2, 3 and 4 offspring compared with trios (*P* values were 0.022, 2.478 × 10^-5^ and 7.396 × 10^-7^ at depth 6x; 0.001, 3.698 × 10^-7^ and 1.822 × 10^-5^ at depth 10x). For SNP with MAF <5 % at depth 2x, the *p* values are 0.014, 4.43 × 10^-6^ and 3.698 × 10^-7^ as including 2, 3 and 4 offspring compared with trios (*P* values were 0.0001, 7.396 × 10^-7^ and 3.698 × 10^-7^ at depth 6x; 3.698 × 10^-7^, 3.698 × 10^-7^ and 3.698 × 10^-7^ at depth 10x). In addition, we also applied “TrioCaller” 100 times by randomly selected a child to form a trio and all the other children as independent individuals for all 80 families in the first simulation and took the consensus genotype calls from 100 results. Additional file 1: Figure S2 shows the genotype mismatch rate of heterozygous calls were 0.0476, 0.0117 and 0.0035 at depth 2x, 6x and 10x, respectively. There is no improvement of genotype mismatch rate by taking consensus genotype calls from 100 results as compared with the result from TrioCaller. The second simulation scheme considered alignment and experimental errors also targeted on the first purpose. Additional file 1: Table S2 shows the genotype mismatch rate of heterozygous calls. In general, GATK has high genotype mismatch rate, especially with depth 5x (16.4 %) and 10x (2.7 %) and our proposed method greatly outperformed the results from GATK. When all three offspring were all considered in our algorithm, the genotyping errors of heterozygous SNPs reduced from 16.4 % and 2.7 % to 0.9 % and 0.4 % at 5x and 10x coverage, respectively. Genotype mismatch rate will keep decreasing when adding more offspring when using our proposed method, especially at low depth 5x. The genotype discordance error rate can be reduced from 0.92 % to 0.84 % to 0.77 % by considering one, two and three offspring in each family at 5x coverage. Furthermore, we selected five members from the pedigree in Additional file 1: Figure S1 to form a complex family structure (with three generations) and Additional file 1: Table S2 shows our proposed method can still improve the genotype accuracy of heterozygous SNPs (genotype mismatch rate are 0.86, 0.37, 0.24 and 0.25 % at depths 5x, 10x, 20x and 30x, respectively). In addition, GATK considered trio information for genotype calling, the genotype mismatch rate can be reduced from 16.4 to 10.52 %, 2.77 to 1.75 %, 0.45 to 0.36 %, 0.31 to 0.26 % at 5x, 10x, 20x, 30x coverage, respectively.

### Performance of haplotyping

**Fig. 3:**
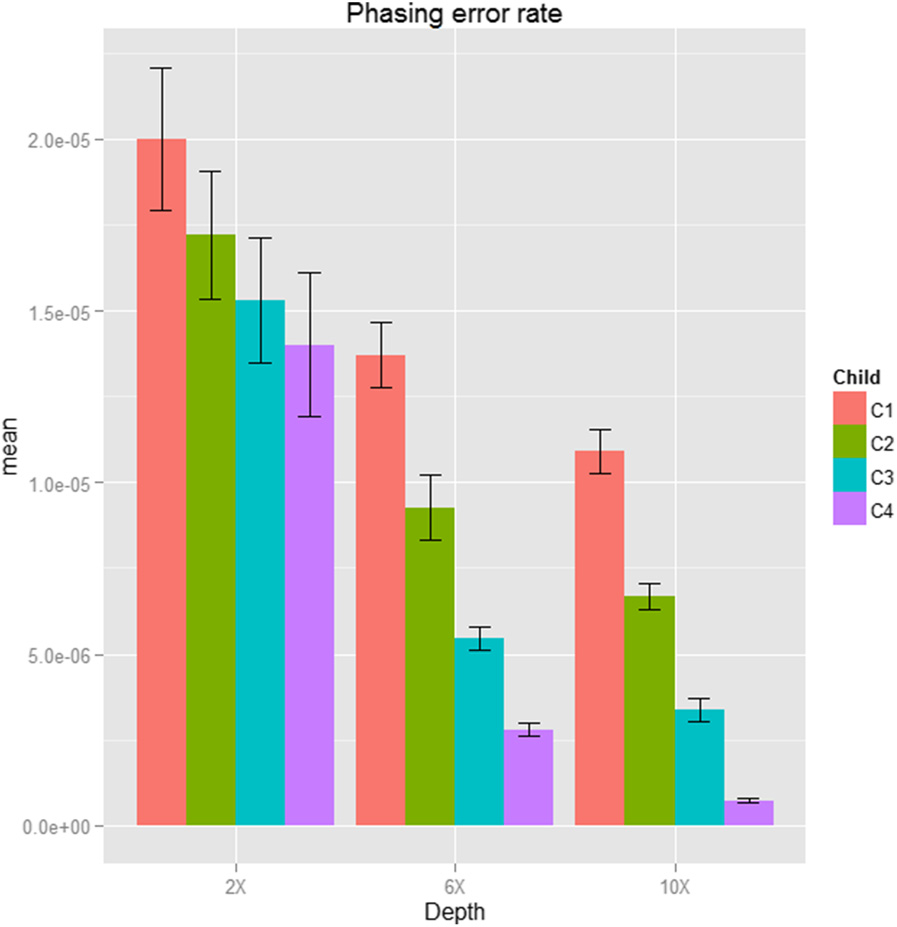
Phasing error rate and standard errors (Simulation I). The phasing rate of sequencing coverage of 2x, 6x and 10x and bases with Phred-scaled quality Q20 (1 % error per-based rate). (C1: trios; C2: nuclear families of two offspring; C3: nuclear families with three offspring and C4: nuclear families of four offspring.)

Haplotype reconstruction plays an important role for follow-up analysis such as genotype imputation, and studying the population history. The phasing error rates were calculated by the mean number of mismatched alleles between reconstructed haplotypes by using our proposed algorithm and haplotypes from simulated data (we assumed the simulated haplotypes was underlying truth). The first simulation results from 12 simulated haplotypes are summarized in Fig. 3 and Additional file 1: Table S3. In summary, at low depth 2x, adding more offspring in each family can keep reducing switch errors. For instance, the phasing error rate can reduce 25 % when all four offspring were taken into consideration compared with trio-based (only considers one offspring). Similar to genotype accuracy, sequencing depth contributed to phasing error rate, but our proposed method still showed its advantage when lowering the phasing error when adding more offspring. The p values of Wilcoxon sun rank test of the phasing error rate of 2, 3 and 4 offspring in the unclear family compared with the result from trios at depth 2x were 0.071, 0.007 and 0.018, respectively (*P* values were 0.0001, 3.698 × 10^-7^ and 3.698 × 10^-7^ at depth 6x; 2.589 × 10^-6^, 3.698 × 10^-7^ and 3.698 × 10^-7^ at depth 10x). Beagle shows perfect phasing in our simulation possibly because there are more miscalled heterozygotes, which are not counted in the calculation of switch errors. In the second simulation, Additional file 1: Table S4 shows our proposed algorithm has better phasing error in 5x and 10x coverage than GATK, but not in 20x and 30x coverage.

### Performance of Mendelian errors

**Fig. 4:**
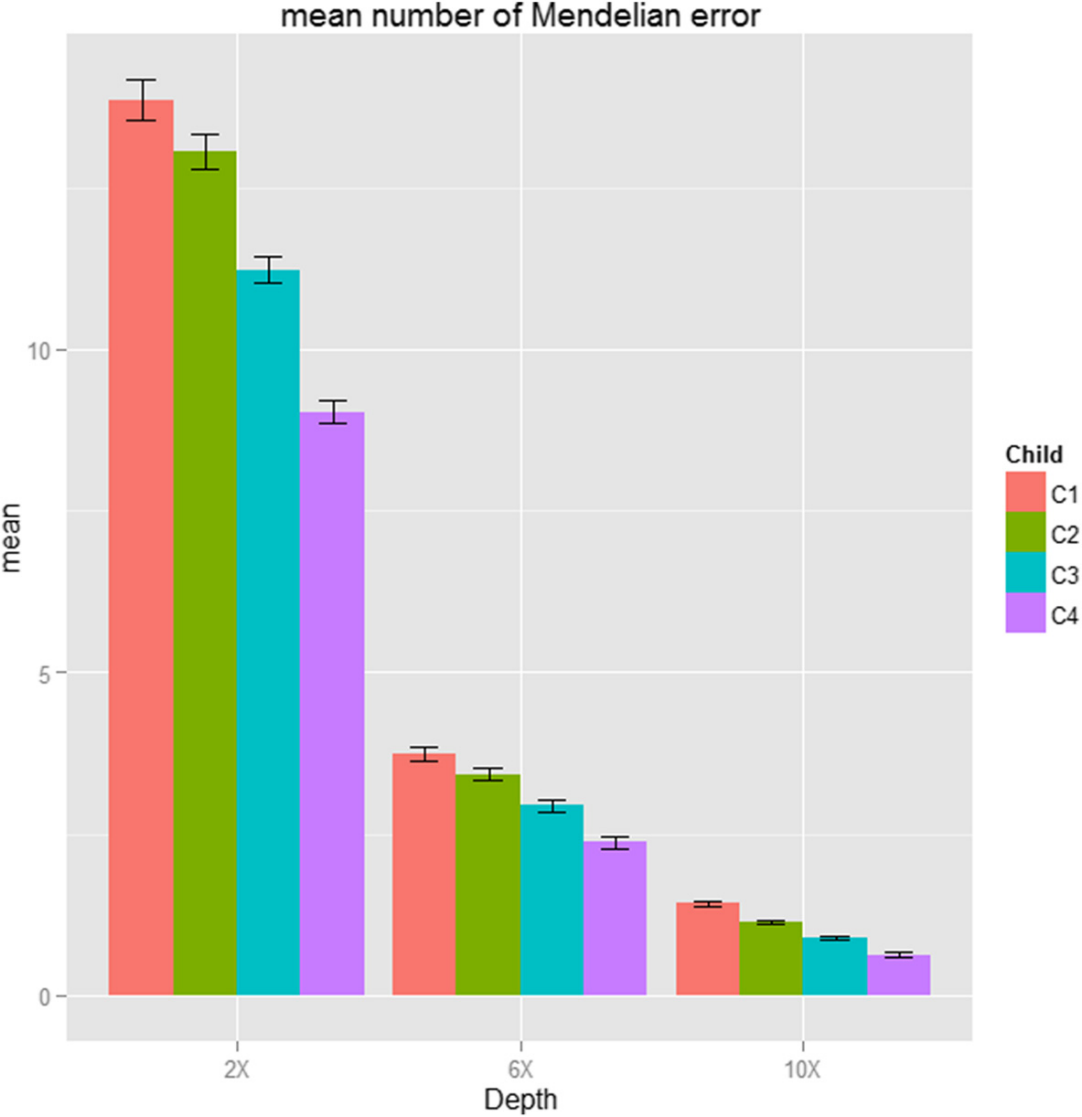
Mendelian error rate and standard errors (Simulation I). The mean number of Mendelian errors for each offspring with sequencing coverage of 2x, 6x and 10x and bases with Phred-scaled quality Q20 (1 % error per-based rate). (C1: trios; C2: nuclear families of two offspring; C3: nuclear families with three offspring and C4: nuclear families of four offspring.)

Since our proposed method considered the family constraint (we considered trio at a time within whole family structure), we can lower the Mendelian errors. We calculated the Mendelian errors by calculating the total number of Mendelian inconsistent genotypes divided by the total number of offspring in simulated data set. In our first simulation study without considering alignment and experimental errors, the mean number of Mendelian errors of our proposed method when considering all four offspring compared with trio-based methods in simulated data can be dropped from 13.86 to 9.04, 3.74 to 2.37 and 1.42 to 0.63 at 2x, 6x and 10x coverage, respectively (see Fig. 4 and Additional file 1: Table S5). The p values of Wilcoxon sun rank test of the phasing error rate of 2, 3 and 4 offspring in the unclear family compared with the result from trios at depth 2x were 0.057, 3.698 × 10^-7^ and 3.698 × 10^-7^, respectively (*P* values were 0.023, 1.664 × 10^-5^ and 3.698 × 10^-7^ at depth 6x; 4.85 × 10^-5^, 1.822 × 10^-5^ and 3.698 × 10^-7^ at depth 10x). The second simulation summarized in Table 1 showed the mean number of Mendelian errors for each offspring with considering alignment and experimental errors. As compared with the results from GATK, our proposed method can reduce the mean number of Mendelian error from 28,213 and 6475 to 718 and 228 at 5x and 10x coverage, respectively. In addition, when adding more offspring into consideration, our algorithm can achieve lower Mendelian errors, especially with low depth 5x: the mean number of Mendelian errors were reduced from 1118 to 962 to 718 when considering one, two and three offspring in each family, respectively.

**Table 1:** Mendelian error rate (Simulation II). The mean number of Mendelian errors for each offspring with sequencing coverage of 5x, 10x. 20x and 30x from our proposed method “FamLDCaller” (FLDC) compared with the results from Genome Analysis Toolkit (GATK). (F3: trios; F4: nuclear families of two offspring; F5: nuclear families with three offspring and F6: complex families with three generations.)

Depth	5		10		20		30	
Method	GATK	FLDC	GATK	FLDC	GATK	FLDC	GATK	FLDC
F3	28,213	1118	6475	483	927	182	628	164
F4	28,213	962	6475	350	927	135	628	126
F5	28,213	718	6475	228	927	94	628	86
F6	33,427	351	7420	99	1161	46	805	39

### Performance of incorporating reference panels

**Fig. 5:**
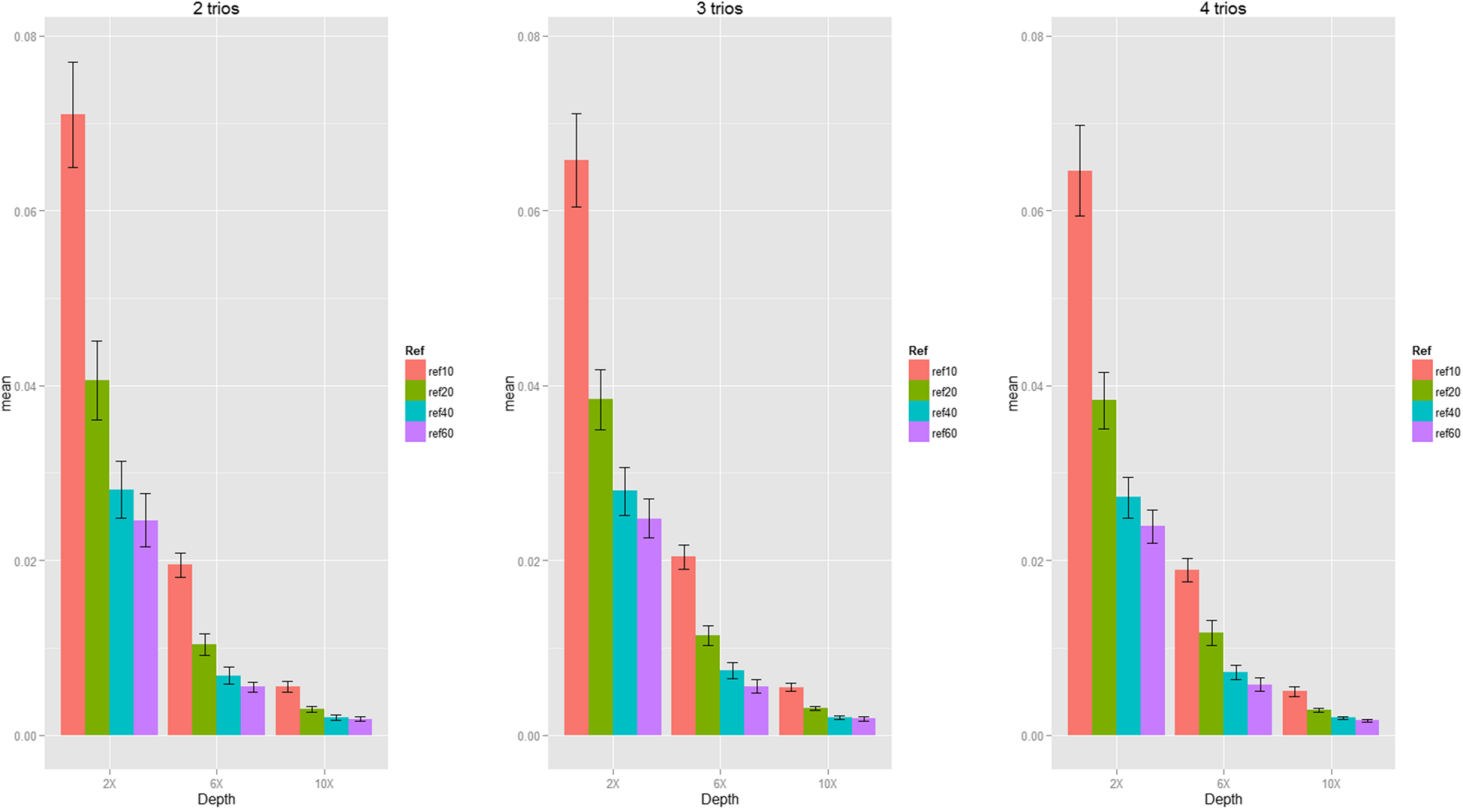
Genotype discordance rate and standard errors of heterozygous calls (Simulation III). The proportion of genotype mismatch rate for heterozygous SNPs with sequencing coverage of 2x, 6x and 10x and bases with Phred-scaled quality Q20 (1 % error per-based rate). (ref10: 10 founders; ref20: 20 founders; ref30: 30 founders and ref40: 40 founders in reference panels from 1000 Genome Project)

**Fig. 6:**
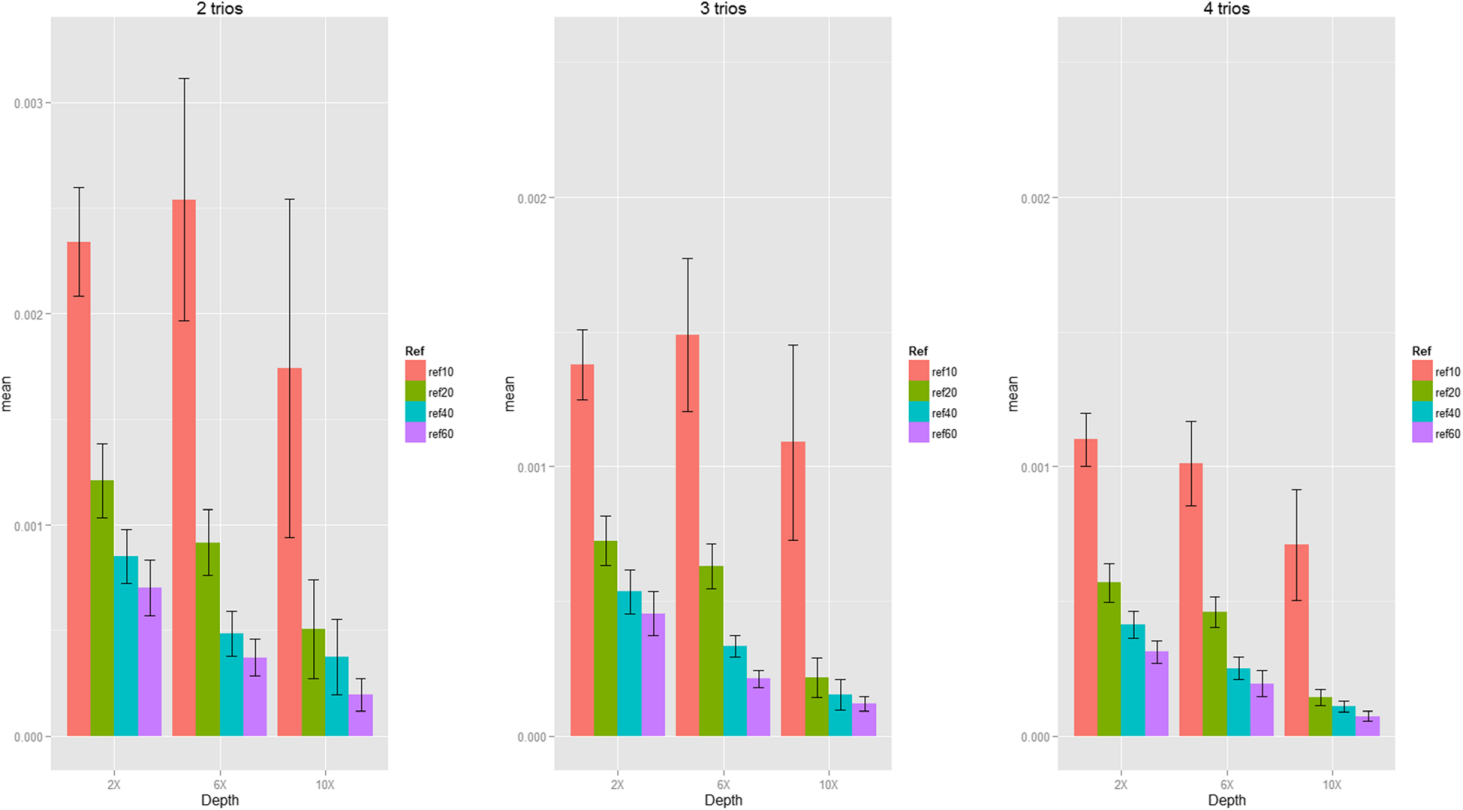
Phasing error rate and standard errors (Simulation III). The phasing error rate for heterozygous SNPs with sequencing coverage of 2x, 6x and 10x and bases with Phred-scaled quality Q20 (1 % error per-based rate). (ref10: 10 founders; ref20: 20 founders; ref30: 30 founders and ref40: 40 founders in reference panels from 1000 Genome Project)

**Fig. 7:**
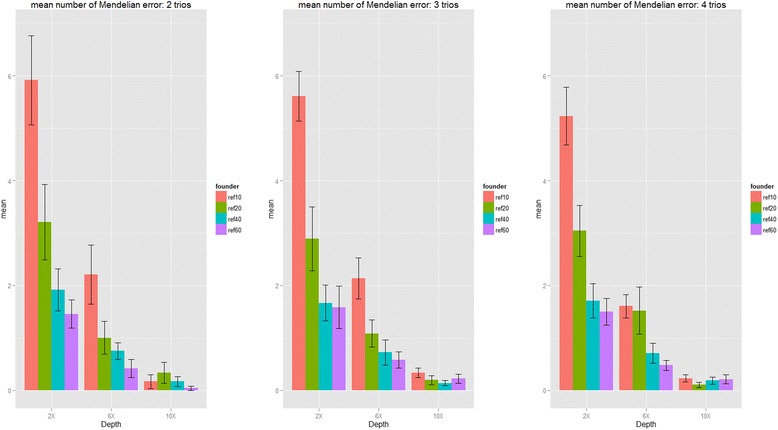
Mendelian error rate and standard errors (Simulation III). The mean number of Mendelian errors for each offspring with sequencing coverage of 2x, 6x and 10x and bases with Phred-scaled quality Q20 (1 % error per-based rate). (ref10: 10 founders; ref20: 20 founders; ref30: 30 founders and ref40: 40 founders in reference panels from 1000 Genome Project)

Next, we proceeded to evaluate the genotype mismatch rates, phasing errors and Mendelian errors by incorporating external references when sequencing data with small sample sizes for our second purpose (see the simulation results summarized in Figs. 5, 6 and 7 and Additional file 1: Table S6 to Table S8). In summary, for limited number of sequenced samples, by incorporating external references our proposed algorithm can also provide the accurate genotypes, and reduce the phasing errors and Mendelian errors. For example, the genotype mismatch rates dropped from 7 to 4 % to 2.8 to 2.4 %, the phasing error rates dropped from 0.2 to 0.12 % to 0.08 to 0.07 %, and the mean number of Mendelian errors dropped from 5.92 to 3.21 to 1.92 to 1.46 when incorporating 10, 20, 40 and 60 founders from 1000 Genome Project for 2 sequenced trios at 2x coverage. Sequencing depth is also a key factor for genotype accuracy, and we found that increasing the number of external references (founders) can be a good way to compensate the depth: the genotype mismatch rate at coverage 2x incorporated by 60 founders is 2.4 %, which is similar to the genotype mismatch rate at coverage 6x incorporated by 10 founders (~2 %); the genotype mismatch rate at coverage 6x incorporated by 60 founders is 0.55 %, which is similar to the genotype mismatch rate at coverage 10x incorporated by ten founders (0.56 %).

### Performance on real data

We applied our methods to an ongoing sequencing project, which has a total of 1339 samples and includes 623 families with an average depth 10x from the Minnesota Center for Twin and Family Research [29]. The sequencing experiment was conducted using Illumina HiSeq 2000 at the University of Michigan. We focused on 1,880,175 sites on chromosome 20 and calculated the mismatch rate between the called genotypes from our method and the available genotypes from accurate DNA microarray chips. Then, we compared the mismatch rate using our methods with that using other existing method Beagle [30] and Thunder [18]. All genotype discordance of stratified analysis were summarized in Table 2. For all SNPs, the genotype mismatch rate of our method with 100 rounds, Beagle and Thunder are 0.000798, 0.001049 and 0.001158; For heterozygous SNPs, the genotype mismatch rate of our method with 100 rounds, Beagle and Thunder are 0.01552, 0.00195 and 0.002281. We will continue our investigation when more sequence data are available. For SNPs with maf <5 %, the genotype mismatch rate of our method with 100 rounds, Beagle and Thunder are 0.003503, 0.002669 and 0.004161. Specifically, we also investigated few small regions on chromosome 20 using different states (200, 400 and 600), as a result, the genotype mismatch rate for heterozygous calls was reduced from 0.001224 to 0.001035 to 0.001016 when 200, 400 and 600 states were used. When comparing with Beagle, the genotype mismatch rate for SNPs with maf <5 % from our method using 200 states is comparable with that from Beagle.

**Table 2:** Genotype discordance rate of all SNPs, heterozygous SNPs and SNPs with maf <5 % in the real data analysis of Minnesota Center for Twin and Family Research

	All SNPs	Heterozygotes	SNPs with maf <5 %
FamLDCaller (100 states)	0.00080	0.0016	0.0035
FamLDCaller (200 states)	0.00066	0.0012	0.0025
FamLDCaller (400 states)	0.00056	0.0010	0.0018
FamLDCaller (600 states)	0.00059	0.0010	0.0018
Beagle	0.00105	0.0019	0.0027
Thunder (200 states)	0.00116	0.0023	0.0042

We also applied our method to the 1000 Genomes Project (www.1000genomes.org) on deep sequenced trios for our second purpose to incorporate external panels when analyzing family-based sequencing data with small sample size. There are two trios with one trio from CEU and the other from YRI. These two trios have been genotyped on OMNI chip. For CEU trio, the genotype mismatch rates are $1.48\times {10}^{-3}$ and $1.78\times {10}^{-3}$ for all and heterozygous SNPs, respectively; for YRI trio, the genotype mismatch rate are $1.89\times {10}^{-3}$ and $2.35\times {10}^{-3}$ for all and heterozygous SNPs, respectively. It implies that our method can achieve reasonable accuracy in genotype calling. TrioCaller or GATK does not have such functionality to incorporate external panels.

## Conclusions and discussions

In this study, we proposed a computationally efficient algorithm to infer genotypes by considering multiple offspring in family-based sequencing data. Our proposed method outperforms existing programs such as TrioCaller, GATK, and Beagle in general families with multiple offspring at low to modest sequencing coverage. In the simulation studies, we showed our proposed algorithm can obtain more accurate genotype calls, lower phasing errors and Mendelian errors, compared with the methods that only ignore family or LD information. In addition, our proposed algorithm provides a function to incorporate the external panels when only a small number of family samples (e.g. 2–4 trios) are sequenced. We showed that our method can achieve satisfying results for small-scale studies using external reference panels.

Comparing to existing methods, one advantage of our implementation is that it allows external reference panels as input. Although sequencing cost has dropped significantly in the past few years, it is still not practical to perform large-scale whole-genome sequencing studies for most laboratories; typically one or a few families are sequenced as a pilot study. LD-aware approaches are not working appropriately if only a small number of families are sequenced because the number of independent samples limits state space. Using external phased haplotypes from a similar population, our algorithm can efficiently construct state space and infer sequence samples. Most existing software do not provide such functionality and are not applicable to small-scale studies, while our implementation can directly take external phased haplotypes in VCF format. We suggest that this function can be used when an external panel with similar LD structures of study population are available.

The computational cost of our method is comparable to LD-aware methods for unrelated individuals. Practically, methods (e.g. TrioCaller) on trio data can be applied on general families. For each nuclear family, we can randomly pick a child to form a trio with the parents and treat the other children as unrelated individuals. A child is randomly chosen to be included in the trio in each Markov chain Monte Carlo (MCMC) iteration. Each child will be included in the trio in certain iterations. The haplotypes sampled in each iteration are merged to generate consensus haplotypes while minimizing crossovers over all sampled haplotypes. However, the convergence of such a strategy might be slow because only one child is included in the trio in each round, and the inferred haplotypes of the parents are not able to reflect the actual transmission to each child, particularly in a region where family recombination occurs. For convergence rates of our algorithm, we tested different number of rounds 20, 40, 60 and 80 compared with 100 rounds that we used in the first simulation study. For 80 nuclear families with four offspring were all included, the genotype mismatch rate of heterozygous calls at depth 2x were 0.0459, 0.0418, 0.0405, 0.04007 and 0.0398 when using 20, 40, 60, 80 and 100 rounds. In addition, the genotype mismatch rate of heterozygous calls at higher depth 10x were 0.00161, 0.00152, 0.00149, 0.00145 and 0.0145 when using 20, 40, 60, 80 and 100 rounds. In summary, the genotype converges faster at high depth, and increasing the number of rounds can achieve more accurate genotypes but may require more computational cost. Our method also works for complex family such as the pedigree has three generations, and the complexity of the pedigree will affect the convergence rate, but not much because we form all possible trio combination from the pedigree in each iteration, which can only affect the computing time. Mendelian errors are expected to be elevated. In our implementation, each child is modeled separately in each MCMC round. Our approach duplicates parents for each child after loading the data. Hence, a nuclear family with *n* children will be split into *n* trios (parents are duplicated *n-1* times). We consider these *n* trios to be independent in each round. Each pair of duplicated parents will be sampled and consensus haplotypes will be generated at the end of the process. Thus, all children will contribute to parents and transmission information is stored in each trio. Our strategy balances the computational and statistical complexity. Roughly, computation cost increases linearly with the average number of offspring in each family.

To further refine the method, reduce Mendelian errors, and accurately detect recombination points, a more statistically rigorous method could be implemented to jointly model the whole family. However, the computation is not feasible in practice because the state space increases exponentially. Advanced models are needed to combine both LD information and genetic inheritance in a rigorous and efficient way. We anticipate that phasing quality can be further improved -- we will explore this direction in future. We note there are ongoing improvements from other tools such as GATK, BEAGLE, IMPUTE2 and SHAPEIT. Those improvements can be potentially complementary to our approach. Nevertheless, we provide a unique and practical tool to infer genotype and phasing for general families. A comprehensive study of family-based genotype calling methods is beyond the scope of the current study and will be explored elsewhere.

Our method also provides some benefits for downstream association analysis. The proposed process is stochastic. Sampled haplotypes for each individual are drawn in each round and a consensus haplotype is generated for final output. This process provides extra sampling information (uncertainty of genotype calling) that can be used in downstream association analyses. For example, we can perform multiple association analyses using sampled haplotypes and summarize test statistics using a multiple imputation framework. Existing LD-aware methods usually generate final consensus haplotypes without such options.

We have implemented our methods efficiently in a C++ program FamLDCaller, which is available from http://www.pitt.edu/~wec47/famldcaller.html. The program can take standard VCF and pedigree files as input. Background information and tutorial examples are also provided to facilitate researchers who are not familiar with genotype calling for family-based sequencing data. We hope the packages can be useful to the processing of family-based sequencing data.

To summarize, we present a computational tool for genotype calling and haplotype inference in complex families. This tool is complementary to existing methods and can be valuable to many ongoing family-based sequencing project.

## Additional files

Additional file 1: Table S1. Genotype mismatch rate of heterozygous calls and SNPs with maf <5 % (Simulation I). **Table S2.** Genotype discordance rate of heterozygous calls (Simulation II). **Table S3.** Phasing error rate (Simulation I). **Table S4.** Phasing error rate (Simulation II). **Table S5.** Mendelian error rate (Simulation I). **Table S6.** Genotype discordance rate of heterozygous calls (Simulation III). **Table S7.** Phasing error rate (Simulation III). **Table S8.** Mendelian error rate (Simulation III). **Figure S1.** Pedigree of each family in second simulation scheme. **Figure S2.** Genotype mismatch rate of heterozygous calls (Simulation I). C1: Trios result summarized by ranodmly selected a child to form a trio and all the other children as independent individuals for all 80 families with 100 repeats; C2: nuclear families of two offspring; C3: nuclear families with three offspring and C4: nuclear families of four offspring. (DOCX 1452 kb)
