# Supplementary material for: A computational method for genotype calling in family-based sequencing data
Source: BMC Bioinformatics. 2016 Jan 16;17:37. doi: 10.1186/s12859-016-0880-5 (PMC4715317; doi:10.1186/s12859-016-0880-5)
Supplement: Additional file 1: Table S1. — Genotype mismatch rate of heterozygous calls and SNPs with maf <5 % (Simulation I). Table S2. Genotype discordance rate of heterozygous calls (Simulation II). Table S3. Phasing error rate (Simulation I). Table S4. Phasing error rate (Simulation II). Table S5. Mendelian error rate (Simulation I). Table S6. Genotype discordance rate of heterozygous calls (Simulation III). Table S7. Phasing error rate (Simulation III). Table S8. Mendelian error rate (Simulation III). Figure S1. Pedigree of each family in second simulation scheme. Figure S2. Genotype mismatch rate of heterozygous calls (Simulation I). C1: Trios result summarized by ranodmly selected a child to form a trio and all the other children as independent individuals for all 80 families with 100 repeats; C2: nuclear families of two offspring; C3: nuclear families with three offspring and C4: nuclear families of four offspring. (DOCX 1452 kb) [file 12859_2016_880_MOESM1_ESM.docx]

**Supplementary Materials for**

**A Computational Method for Genotype Calling in Family-based Sequencing Data**

### Lun-Ching Chang, Bingshan Li, Zhou Fang, Scott Vrieze, Matt McGue, William G. Lacono, George C. Tseng, and Wei Chen*

**Supplementary Material**

Running Parameters of GATK and Beagle

**GATK**: jar GenomeAnalysisTK.jar -R ref.fa –T UnifiedGenotyper –I sample1.bam –I sample2.bam –o GATK.vcf

**GATK Trio**: jar GenomeAnalysisTK.jar -T PhaseByTransmission -R ref.fa --pedigreeValidationType SILENT -V GATK.vcf -ped input.ped -o output.GATKtrio.vcf

**Beagle**: java -jar ./beagle.12Oct15.b2c.jar ped=input.ped gl=input.vcf out=output.beagle4

**Supplementary Tables and Figures**

**Supplementary Table 1. Genotype mismatch rate of heterozygous calls and SNPs with maf < 5% (Simulation I).** The proportion of genotype mismatch rate for heterozygous SNPs and SNPs with minor allele frequency (MAF) < 5% with sequencing coverage of 2x, 6x and 10x and bases with Phred-scaled quality Q20 (1% error per-based rate).

|  |  | 2X | 6X | 10X |
| --- | --- | --- | --- | --- |
| Het | 80 trios, 240 unrelated (TrioCaller) | 0.0448 | 0.0088 | 0.0026 |
|  | 80 nuclear families (two offspring), 160 unrelated | 0.0438 | 0.0076 | 0.0020 |
|  | 80 nuclear families (three offspring), 80 unrelated | 0.0419 | 0.0064 | 0.0015 |
|  | 80 nuclear families (four offspring) | 0.0394 | 0.0052 | 0.0011 |
|  | Beagle (considering pedigree) | 0.0519 | 0.0070 | 0.0022 |
| SNPs with MAF < 5% | 80 trios, 240 unrelated (TrioCaller) | 0.0093 | 0.0020 | 0.0006 |
|  | 80 nuclear families (two offspring), 160 unrelated | 0.0089 | 0.0017 | 0.0005 |
|  | 80 nuclear families (three offspring), 80 unrelated | 0.0084 | 0.0015 | 0.0004 |
|  | 80 nuclear families (four offspring) | 0.0079 | 0.0011 | 0.0003 |
|  | Beagle (considering pedigree) | 0.0094 | 0.0015 | 0.0005 |

**Supplementary Table 2. Genotype discordance rate of heterozygous calls (Simulation II).** The proportion of genotype mismatch rate for heterozygous SNPs with sequencing coverage of 5x, 10x. 20x and 30x from our proposed method “FamLDCaller” (FLDC) compared with the results from Genome Analysis Toolkit (GATK). (F3: trios; F4: nuclear families of two offspring; F5: nuclear families with three offspring and F6: complex families with three generations.)

| **Depth** | **5** | | **10** | | **20** | | **30** | |
| --- | --- | --- | --- | --- | --- | --- | --- | --- |
| **Method** | **GATK** | **FLDC** | **GATK** | **FLDC** | **GATK** | **FLDC** | **GATK** | **FLDC** |
| **F3** | 0.1640 | 0.0092 | 0.0277 | 0.0042 | 0.0045 | 0.0026 | 0.0031 | 0.0026 |
| **F4** | 0.1640 | 0.0084 | 0.0277 | 0.0037 | 0.0045 | 0.0025 | 0.0031 | 0.0026 |
| **F5** | 0.1640 | 0.0077 | 0.0277 | 0.0032 | 0.0045 | 0.0024 | 0.0031 | 0.0025 |
| **F6** | 0.1638 | 0.0086 | 0.0276 | 0.0037 | 0.0045 | 0.0024 | 0.0031 | 0.0025 |

**Supplementary Table 3. Phasing error rate (Simulation I).** The phasing rate of sequencing coverage of 2x, 6x and 10x and bases with Phred-scaled quality Q20 (1% error per-based rate).

|  |  | 2X | 6X | 10X |
| --- | --- | --- | --- | --- |
| BE = 20 | 80 trios, 240 unrelated (TrioCaller) | 2e-05 | 1.37e-05 | 1.09e-05 |
|  | 80 nuclear families (two offspring), 160 unrelated | 1.72e-05 | 9.27e-06 | 6.68e-06 |
|  | 80 nuclear families (three offspring), 80 unrelated | 1.53e-05 | 5.45e-06 | 3.36e-06 |
|  | 80 nuclear families (four offspring) | 1.4e-05 | 2.8e-06 | 7.22e-07 |
|  | Beagle (considering pedigree) | 0 | 0 | 0 |

**Supplementary Table 4. Phasing error rate (Simulation II).** The phasing rate of sequencing coverage of 2x, 6x and 10x and bases with Phred-scaled quality Q20 (1% error per-based rate). GATK considered trio information for phasing.

| **Depth** | **5** | | **10** | | **20** | | **30** | |
| --- | --- | --- | --- | --- | --- | --- | --- | --- |
| **Method** | **GATK** | **FLDC** | **GATK** | **FLDC** | **GATK** | **FLDC** | **GATK** | **FLDC** |
| **F3** | 0.0285 | 0.00288 | 0.0625 | 0.00268 | 0.00059 | 0.00263 | 0.00023 | 0.00264 |

**Supplementary Table 5. Mendelian error rate (Simulation I).** The mean number of Mendelian errors for each offspring with sequencing coverage of 2x, 6x and 10x and bases with Phred-scaled quality Q20 (1% error per-based rate).

|  |  | 2X | 6X | 10X |
| --- | --- | --- | --- | --- |
| BE = 20 | 80 trios, 240 unrelated (TrioCaller) | 13.86 | 3.74 | 1.42 |
|  | 80 nuclear families (two offspring), 160 unrelated | 13.06 | 3.42 | 1.14 |
|  | 80 nuclear families (three offspring), 80 unrelated | 11.23 | 2.95 | 0.89 |
|  | 80 nuclear families (four offspring) | 9.04 | 2.37 | 0.63 |
|  | Beagle (considering pedigree) | 14.39 | 3.23 | 1.06 |

**Supplementary Table 6. Genotype discordance rate of heterozygous calls (Simulation III).** The proportion of genotype mismatch rate for heterozygous SNPs with sequencing coverage of 2x, 6x and 10x and bases with Phred-scaled quality Q20 (1% error per-based rate) using different number of founders (10, 20, 40 and 60) as reference panels from 1,000 Genome Project when analyzing simulated family-based sequencing data set with 2, 3 and 4 trios.

|  | reference (# of founders) | 10 | 20 | 40 | 60 |
| --- | --- | --- | --- | --- | --- |
|  |  | 2 trios | | | |
| BE = 20 | 2X | 0.0710 | 0.0406 | 0.0281 | 0.0246 |
|  | 6X | 0.0195 | 0.0104 | 0.0068 | 0.0055 |
|  | 10X | 0.0056 | 0.0030 | 0.0020 | 0.0019 |
|  |  | 3 trios | | | |
| BE = 20 | 2X | 0.0658 | 0.0384 | 0.0279 | 0.0248 |
|  | 6X | 0.0204 | 0.0114 | 0.0074 | 0.0056 |
|  | 10X | 0.0054 | 0.0031 | 0.0021 | 0.0019 |
|  |  | 4 trios | | | |
| BE = 20 | 2X | 0.0646 | 0.0383 | 0.0272 | 0.0239 |
|  | 6X | 0.0189 | 0.0117 | 0.0072 | 0.0058 |
|  | 10X | 0.0050 | 0.0029 | 0.0020 | 0.0017 |

**Supplementary Table 7. Phasing error rate (Simulation III).** The phasing error rate for heterozygous SNPs with sequencing coverage of 2x, 6x and 10x and bases with Phred-scaled quality Q20 (1% error per-based rate) using different number of founders (10, 20, 40 and 60) as reference panels from 1,000 Genome Project when analyzing simulated family-based sequencing data set with 2, 3 and 4 trios.

|  | Reference (# of founders) | 10 | 20 | 40 | 60 |
| --- | --- | --- | --- | --- | --- |
|  |  | 2 trios | | | |
| BE = 20 | 2X | 0.0023 | 0.0012 | 0.0009 | 0.0007 |
|  | 6X | 0.0025 | 0.0009 | 0.0005 | 0.0004 |
|  | 10X | 0.0017 | 0.0005 | 0.0004 | 0.0002 |
|  |  | 3 trios | | | |
| BE = 20 | 2X | 0.0014 | 0.0007 | 0.0005 | 0.0005 |
|  | 6X | 0.0015 | 0.0006 | 0.0003 | 0.0002 |
|  | 10X | 0.0011 | 0.0002 | 0.0002 | 0.0001 |
|  |  | 4 trios | | | |
| BE = 20 | 2X | 0.0011 | 0.0006 | 0.0004 | 0.0003 |
|  | 6X | 0.0010 | 0.0005 | 0.0003 | 0.0002 |
|  | 10X | 0.0007 | 0.0001 | 0.0001 | 7.43e-05 |

**Supplementary Table 8. Mendelian error rate (Simulation III).** The mean number of Mendelian errors for each offspring with sequencing coverage of 2x and 6x and bases with Phred-scaled quality Q20 (1% error per-based rate) using different number of founders (10, 20, 40 and 60) as reference panels from 1,000 Genome Project when analyzing simulated family-based sequencing data set with 2, 3 and 4 trios.

|  | Reference (# of founders) | 10 | 20 | 40 | 60 |
| --- | --- | --- | --- | --- | --- |
|  |  | 2 trios | | | |
| BE = 20 | 2X | 5.9 | 3.2 | 1.9 | 1.5 |
|  | 6X | 2.2 | 1.0 | 0.8 | 0.4 |
|  |  | 3 trios | | | |
| BE = 20 | 2X | 5.6 | 2.6 | 1.7 | 1.6 |
|  | 6X | 2.1 | 1.1 | 0.7 | 0.6 |
|  |  | 4 trios | | | |
| BE = 20 | 2X | 5.2 | 3.0 | 1.7 | 1.5 |
|  | 6X | 1.6 | 1.5 | 0.7 | 0.5 |

**Supplementary Figure 1.** Pedigree of each family in second simulation scheme.


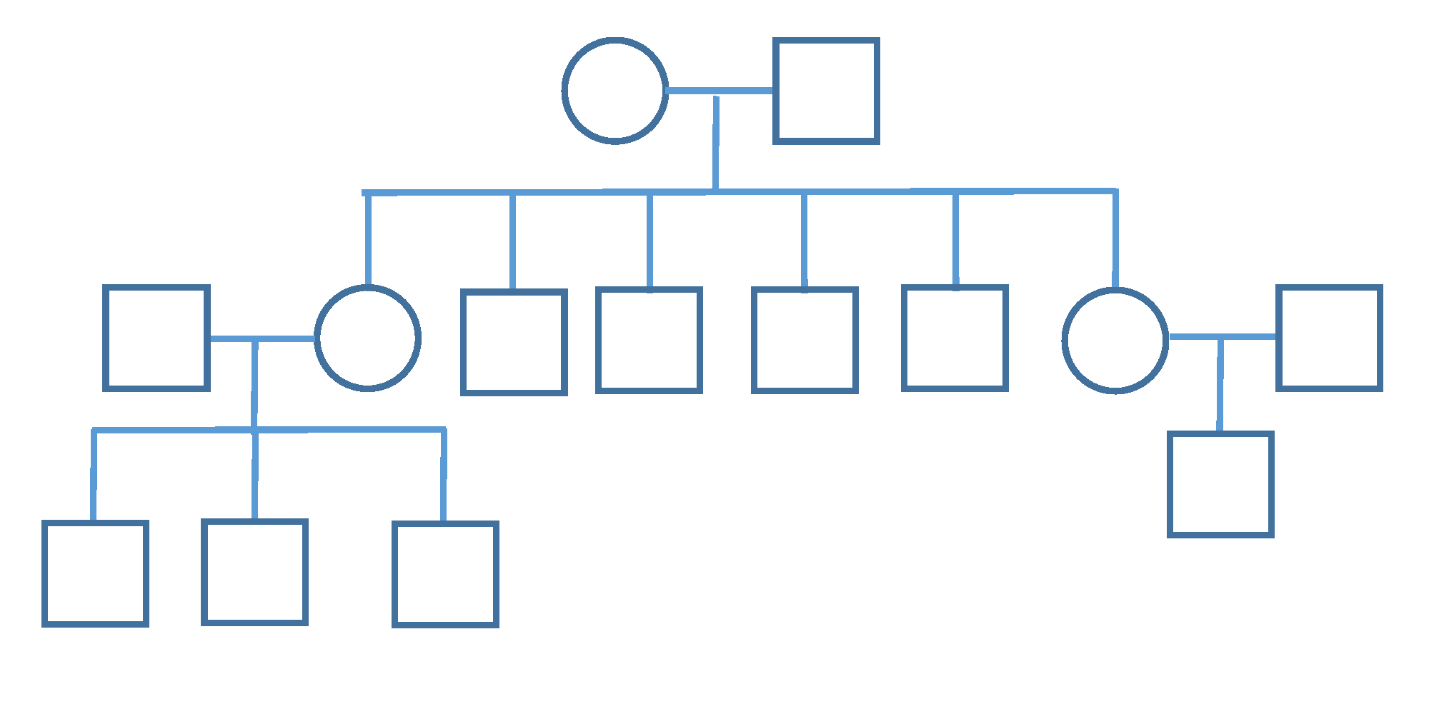


**Supplementary Figure 2.** Genotype mismatch rate of heterozygous calls (Simulation I’).

**
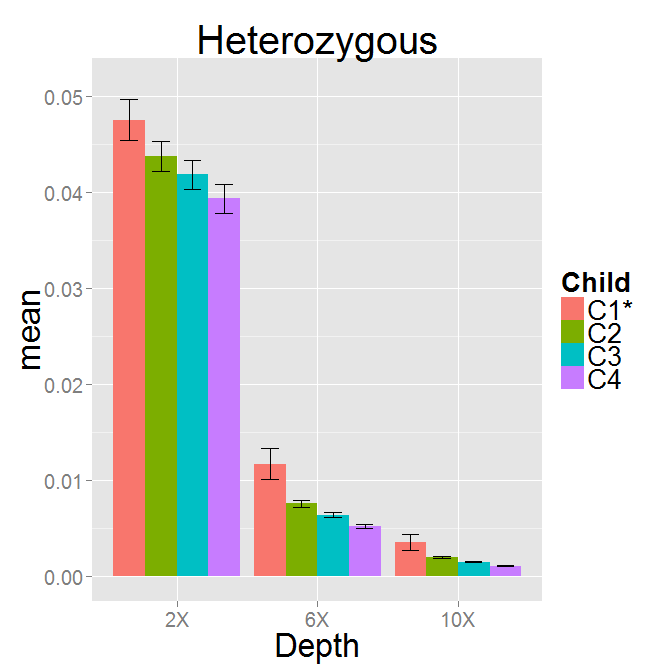
**
